# Supplementary material for: A fault-intrusion-tolerant system and deadline-aware algorithm for scheduling scientific workflow in the cloud
Source: PeerJ Comput Sci. 2021 Nov 2;7:e747. doi: 10.7717/peerj-cs.747 (PMC8576554; doi:10.7717/peerj-cs.747)
Supplement: Supplemental Information 6 [file peerj-cs-07-747-s006.zip › Myproject1.1/docs/org/cloudbus/cloudsim/class-use/UtilizationModel.html]

Uses of Interface org.cloudbus.cloudsim.UtilizationModel


JavaScript is disabled on your browser.


- Overview
- Package
- Class
- Use
- Tree
- Deprecated
- Index
- Help

- Prev
- Next

- Frames
- No Frames

- All Classes

## Uses of Interface org.cloudbus.cloudsim.UtilizationModel

- Packages that use UtilizationModel

  | Package | Description |
  | --- | --- |
  |  |  |
  | --- | --- |
  | org.cloudbus.cloudsim |  |
  | org.cloudbus.cloudsim.network.datacenter |  |
- - ### Uses of UtilizationModel in org.cloudbus.cloudsim

    Classes in org.cloudbus.cloudsim that implement UtilizationModel

    | Modifier and Type | Class and Description |
    | --- | --- |
    |  |  |
    | --- | --- |
    | `class` | `UtilizationModelFull` The UtilizationModelFull class is a simple model, according to which a Cloudlet always utilize all the available CPU capacity. |
    | `class` | `UtilizationModelNull` The UtilizationModelNull class is a simple model, according to which a Cloudlet always require zero capacity. |
    | `class` | `UtilizationModelPlanetLabInMemory` The Class UtilizationModelPlanetLab. |
    | `class` | `UtilizationModelStochastic` The UtilizationModelStochastic class implements a model, according to which a Cloudlet generates random CPU utilization every time frame. |

    Methods in org.cloudbus.cloudsim that return UtilizationModel

    | Modifier and Type | Method and Description |
    | --- | --- |
    |  |  |
    | --- | --- |
    | `UtilizationModel` | Cloudlet.`getUtilizationModelBw()` Gets the utilization model bw. |
    | `UtilizationModel` | Cloudlet.`getUtilizationModelCpu()` Gets the utilization model cpu. |
    | `UtilizationModel` | Cloudlet.`getUtilizationModelRam()` Gets the utilization model ram. |

    Methods in org.cloudbus.cloudsim with parameters of type UtilizationModel

    | Modifier and Type | Method and Description |
    | --- | --- |
    |  |  |
    | --- | --- |
    | `void` | Cloudlet.`setUtilizationModelBw(UtilizationModel utilizationModelBw)` Sets the utilization model bw. |
    | `void` | Cloudlet.`setUtilizationModelCpu(UtilizationModel utilizationModelCpu)` Sets the utilization model cpu. |
    | `void` | Cloudlet.`setUtilizationModelRam(UtilizationModel utilizationModelRam)` Sets the utilization model ram. |

    Constructors in org.cloudbus.cloudsim with parameters of type UtilizationModel

    Constructor and Description

    `Cloudlet(int cloudletId,
    long cloudletLength,
    int pesNumber,
    long cloudletFileSize,
    long cloudletOutputSize,
    UtilizationModel utilizationModelCpu,
    UtilizationModel utilizationModelRam,
    UtilizationModel utilizationModelBw)`

    Allocates a new Cloudlet object.

    `Cloudlet(int cloudletId,
    long cloudletLength,
    int pesNumber,
    long cloudletFileSize,
    long cloudletOutputSize,
    UtilizationModel utilizationModelCpu,
    UtilizationModel utilizationModelRam,
    UtilizationModel utilizationModelBw,
    boolean record)`

    Allocates a new Cloudlet object.

    `Cloudlet(int cloudletId,
    long cloudletLength,
    int pesNumber,
    long cloudletFileSize,
    long cloudletOutputSize,
    UtilizationModel utilizationModelCpu,
    UtilizationModel utilizationModelRam,
    UtilizationModel utilizationModelBw,
    boolean record,
    java.util.List<java.lang.String> fileList)`

    Allocates a new Cloudlet object.

    `Cloudlet(int cloudletId,
    long cloudletLength,
    int pesNumber,
    long cloudletFileSize,
    long cloudletOutputSize,
    UtilizationModel utilizationModelCpu,
    UtilizationModel utilizationModelRam,
    UtilizationModel utilizationModelBw,
    java.util.List<java.lang.String> fileList)`

    Allocates a new Cloudlet object.
  - ### Uses of UtilizationModel in org.cloudbus.cloudsim.network.datacenter

    Constructors in org.cloudbus.cloudsim.network.datacenter with parameters of type UtilizationModel

    Constructor and Description

    `NetworkCloudlet(int cloudletId,
    long cloudletLength,
    int pesNumber,
    long cloudletFileSize,
    long cloudletOutputSize,
    long memory,
    UtilizationModel utilizationModelCpu,
    UtilizationModel utilizationModelRam,
    UtilizationModel utilizationModelBw)`

- Overview
- Package
- Class
- Use
- Tree
- Deprecated
- Index
- Help

- Prev
- Next

- Frames
- No Frames

- All Classes
